# SNAP recipients’ experiences during the 2025 government shutdown: implications for food access

**DOI:** 10.3389/fpubh.2026.1812321

**Published:** 2026-05-01

**Authors:** Elizabeth L. Adams, Roger Figueroa, Melanie K. Bean

**Affiliations:** 1 Department of Exercise Science, Arnold School of Public Health, University of South Carolina, Columbia, SC, United States; 2 Research Center for Child Well-Being, University of South Carolina, Columbia, SC, United States; 3 Division of Nutritional Sciences, College of Human Ecology, Cornell University, Ithaca, NY, United States; 4 Department of Pediatrics, Children’s Hospital of Richmond at Virginia Commonwealth University, Richmond, VA, United States

**Keywords:** food access, food insecurity, government shutdown, nutrition policy, public assistance, safety net, Supplemental Nutrition Assistance Program

## Abstract

**Background:**

The 2025 United States government shutdown (October 1–November 12) resulted in historic disruptions to benefit distribution for the Supplemental Nutrition Assistance Program (SNAP). The purpose of this study was to examine South Carolina SNAP recipients’ perceptions of the 2025 government shutdown and how it impacted their food access.

**Methods:**

South Carolina issued SNAP benefits on November 14, 2025, after a maximum 14-day lapse, for households who typically get their benefits between the 1^st^ and 14^th^ of the month. Caregivers (*n* = 74) in SNAP participating in an ongoing clinical trial were invited to complete a brief online survey between November 19 and 27, 2025 to assess if/how November SNAP benefits were impacted and what strategies were used to access food during this time. Descriptive statistics were applied to survey items. Responses for open ended questions were coded into themes.

**Results:**

Fifty-five caregivers completed the survey (76% response rate). Most (85.5%) reported their November SNAP benefits were impacted. Of those impacted, the most common response was a delay in distribution (80.9%). Some caregivers (43.6%) used new or different strategies to access food during the shutdown, the most common being community food resources (e.g., food banks; 62.5%) and support from friends and family (29.2%). When asked to describe these impacts in detail, caregivers described four main themes: constrained food choices, shift toward different foods, financial coping strategies, and emotional strain.

**Conclusion:**

These findings underscore the importance of continuous SNAP benefit distribution and enacting contingency plans during federal disruptions to prevent future undue hardship.

## Introduction

The Supplemental Nutrition Assistance Program (SNAP) provides monthly food assistance to 41 million individuals nationwide (1) and to about 550,000 individuals in South Carolina (10% of the state’s residents) (2). As a federally-funded safety net program, SNAP depends on Congressional appropriations to administer funding each year (3). On October 1, 2025, Congress had not passed a budget for the new fiscal year, triggering the longest government shutdown in United States history. During previous shutdowns, administrations have used contingency funds, carryover appropriations, and short-term adjustments to ensure SNAP benefits were allocated without disruption (4, 5). Yet, 2025 marked a historic departure from the past.

During the 2025 government shutdown, there was uncertainty around the use of contingency funds to support SNAP (6). As a result, many SNAP beneficiaries experienced a delay and/or partial payments in the month of November (7). These disruptions had far-reaching impacts. News outlets reported on the surge in demand among food pantries, with some food banks reporting a 30–50% increase in the number of people utilizing their resources during this time (8, 9). Unfortunately, food pantries could not meet this demand, as for every one meal that emergency food pantries provide, SNAP provides 9 meals (10). Initial reports also showed the steepest increase in food insecurity rates in 2025 occurred from October to November, rising from 13.3 to 16%, respectively (11).

While initial reports and news stories provide important context on the impacts of this government shutdown, there is a lack of scientific research documenting the perspectives from SNAP recipients themselves regarding their experiences with food access during this unprecedented time. Very limited research exists from a prior government shutdown, when February 2019 SNAP benefits were distributed early in January, creating a longer than usual gap before March benefits (12). Although this work offers important background, the 2025 government shutdown represents a distinct and unprecedented situation in which benefits were delayed rather than issued early. Understanding participant’s perspectives in this specific context is needed to inform policy actions that strengthen SNAP resilience during future financial disruptions. The purpose of this study was to examine perceptions of the 2025 government shutdown among SNAP recipients in South Carolina and how it impacted their benefits and food access.

## Materials and methods

### Participants

This study leveraged an ongoing clinical trial (NCT#06828445) on SNAP fruit and vegetable incentive program use in South Carolina (SC). Participants in the parent trial were recruited from Columbia, SC. Eligibility included being ≥18 years of age, the primary caregiver of a child 2–10 years of age, English speaking, and enrolled in SNAP. At trial enrollment, participants also had to be at risk for household food insecurity (13) and have access to a device that received text messages. As part of the parent trial, participants received brief education about a SNAP fruit and vegetable incentive program in SC, and the intervention group received a free trial and free home delivery of the fruit and vegetable incentive program boxes. Informed consent was obtained prior to data collection.

### Study design

All participants active in the parent trial in November 2025 (*n* = 74) were invited to complete a government shutdown impact survey. This cross-sectional survey was added to capture timely data that could be disseminated rapidly and inform the future findings of the larger trial. For context, SC distributes SNAP benefits on a staggered schedule between the 1^st^ and 19^th^ of the month. October SNAP benefits were distributed as usual. November SNAP benefits were delayed with a distribution date of November 14, 2025 for households with a monthly issuance date between the 1^st^ and 14^th^ of the month (14). This survey was administered between November 19 and November 27, 2025. Participants were sent a text message with the survey link and were compensated with a $10 gift card for survey completion. This study was approved by the University of South Carolina Institutional Review Board (Pro00136502).

### Measures

Items (*n* = 5) in the government shutdown impact survey were developed by the research team (Table 1) who are content experts in this area. Caregivers were asked if their November SNAP benefits were impacted by the government shutdown and how so. Caregivers were then provided with a free response question to detail how the government shutdown impacted their access to food, if at all. Lastly, caregivers were asked if they used new or different ways to access food during the shutdown, and if yes, a free response question was posed to detail these new methods. Demographic information provided on the parent trial’s baseline survey were applied to this subsample. Items included caregiver’s age, gender, race, ethnicity, marital status, education, employment, insurance status, annual household income, and duration of SNAP enrollment.

**Table 1:** Survey items administer as part of the government shutdown impact survey.

Survey item followed by response options
1. Were your November SNAP benefits impacted by the government shutdown?
Yes
No
I was not enrolled in SNAP during this time
2. *(If yes)* How were your November SNAP benefits impacted?
I received November benefits, but they were delayed
I still have NOT received November benefits
Other: ______________
3. How did the government shutdown impact your access to food, if at all?
4. Did you use any new or different ways to get food during the government shutdown?
Yes
No
5. *(If yes)* Please explain the new or different ways you obtained food during the government shutdown.

Supplemental Nutrition Assistance Program (SNAP).

### Data analyses

Descriptive statistics were calculated for categorical and continuous survey items using SAS Version 9.4. Responses for open ended questions were coded into themes. The primary author reviewed all participant responses, developed themes grounded in the data using an inductive approach, and iteratively refined these themes. Example quotes were provided to further describe these topics.

## Results

Of the *N* = 74 caregivers invited to participate, *n* = 55 (74.3%) completed the survey. Caregivers demographics are listed in Table 2.

**Table 2:** Caregiver demographics for South Carolina SNAP recipients surveyed post shutdown (*N* = 55).

Demographics	Value
Age, years (mean ± SD)	34.7 ± 7.7
Female (%)	100.0
Race (%)	
Black or African American	80.0
White	10.9
Native Hawaiian or Pacific Islander	1.8
Other	5.5
Hispanic (%)	7.3
Marital status (%)	
Single	70.9
Married	10.9
Domestic partnership	3.6
Separated/divorced	14.5
Education (%)	
High school degree or less	40.0
Some college or vocational training	29.1
Bachelor’s or associate degree	25.5
Graduate or professional degree	5.5
Employment (%)	
Full time work	34.5
Part time work	18.2
In school	9.1
Unemployed	34.5
Retired	3.6
Insurance (%)	
Medicaid	96.4
Private insurance	1.8
None	1.8
Annual household income (mean ± SD)	$17,911 ± 11,910
Time enrolled in SNAP (%)	
≤6 months	18.2
6–12 months	16.4
>12 months	65.5

Supplemental Nutrition Assistance Program (SNAP).

Almost all caregivers (85.5%) reported their November SNAP benefits were impacted by the government shutdown. Of those affected, 80.9% experienced a delay in the distribution of their November benefits. Others reported they had not received their November benefits at the time of the survey (12.8%), or they received benefits that differed from than their typical amount (4.3%).

When asked to describe the impact of the shutdown on their access to food in detail, *n* = 39 participants provided detailed responses that were categorized into four main themes described below. The remaining participants either described no impacts (*n* = 6; e.g., “I had no problem, because they re-opened [the government] before the date they usually send my benefits.”), reiterated the delay without providing further details (*n* = 3; e.g., “I had to wait for them.”), or did not give sufficient detail to be meaningfully analyzed (*n* = 7; e.g., “Yes” or “A lot”). The first of the four themes to emerge was “constrained food choices”. Participants described purchasing and consuming a lower volume of food during the shutdown.

“I had to buy less to make my budget work.”

The second theme was a “shift toward different foods”. Participants described purchasing different foods that were often lower in cost when they had limited funds.

“I have not been able to get a reliable source of healthy meals.”

The third theme was “financial coping strategies”. Participants described the strategies used to afford food during this time.

“I had to use pay in four to order groceries.”

The final theme was “emotional strain” that characterized caregiver’s increased stress around affording food.

“The thought of not being able to buy things if I ran out…knowing I need my cash for bills etc…the shutdown worried me.”

Themes are further described in Table 3.

**Table 3:** Main themes that emerged when participants (*n* = 55) were asked to detail the impact of the 2025 government shutdown on their food access.

Theme	Description	Example quotes
Constrained food intake	Participants purchased and consumed less food during the government shutdown due to limited funds.	*“I wasn’t able to buy all the food that I needed*.” *“We have literally starved*.”
Shift toward different foods	Some participants mentioned purchasing different foods that were, at times, less healthy and cheaper	*“It made me buy food that would last rather than buying healthy food.*” *“We could not afford certain foods.”*
Financial coping strategies	Participants used financial strategies to be able to afford food during the lapse in SNAP benefits, such as reallocating funds and community food resources	*“I had to do payment arrangements on my bills and use the cash for food instead.”* *“My food access was very impacted. I had to visit two food banks.”*
Emotional strain	Caregivers described the stress and worry they felt around affording food	*“I was afraid that my children would not have enough food to take us through the month.”* *“Stress and worry. I spent my October benefits before I wanted because of the rumors of the SNAP card not working at all for November.”*

When asked if they used new or different strategies to access food during the government shutdown, 43.6% of caregivers reported that they had. The most common strategy was accessing community food resources (e.g., food banks, community sites offering food) (62.5%), followed by support from friends and family (29.2%), and other federal assistance programs (e.g., school meal programs, Women, Infants, and Children [WIC] program) (8.3%).

## Discussion

This study examined SNAP recipients’ perceptions of the 2025 government shutdown and how it impacted their food access in SC. Findings from this study demonstrate the immediate negative consequences felt among SNAP recipients in SC during the 2025 government shutdown. An overwhelming majority of caregivers in this study reported a delay in November SNAP benefits, which constrained the type and quantity of food they were able to purchase and consume. These administrative disruptions extended beyond material hardships and affected psychological well-being, as caregivers reported emotional strain due to uncertainty. These results show even brief administrative disruptions result in delayed SNAP benefit distribution and can precipitate immediate hardship and amplify financial and emotional stress. Collectively, these findings underscore the importance of timely and continuous SNAP benefit distributions during federal disruptions to prevent undue hardship.

Data from this study are consistent with findings from the 2018–2019 government shutdown (12). In 2019, funds from a continuing resolution were used to issue February 2019 SNAP benefits early (i.e., in January), resulting in an unusually long gap between February and March SNAP benefit distributions. Qualitative data documented the increased stress, uncertainty, food insecurity, and loss of trust in the government among SNAP recipients (12). Similar to the present study, participants reallocated funds for other essentials in order to afford food and expressed emotional strain and uncertainty. Collectively, these studies shed light on the hardships experienced when safety net programs are unable to provide the consistent support upon which millions of families rely. A failure to provide this consistent and reliable support can undermine trust in the program and the institutions that administer it. Such erosion of trust could have lasting consequences on program participation, engagement, perceived reliability, and thus effectiveness of health outcomes.

During the November 2025 shutdown, some caregivers in this study reported purchasing cheaper and less nutritious food, out of necessity, which has important implications on diet quality. The high cost of affording nutritious food is cited as one of the most prominent barriers to adequate dietary intake among SNAP recipients (15). Disruptions in SNAP benefits compound the challenges that families already face in consuming a diet that is associated with numerous health benefits and underscore the importance of contingency plans to protect benefit distributions during times of government disruption. During past government shutdowns, contingency reserves, carryover appropriations, and short-term adjustments were used to prevent delays in the distribution of SNAP funds. Moving forward, contingency plans to ensure these strategies are used could include reserve funding triggers where contingency funds are automatically activated when federal appropriations are disrupted, thus allowing states to continue issuing benefits on schedule without last-minute legislative or administrative action.

The limitations of this study include a small sample size and the use of a convenience sample that was part of a larger clinical trial. This sample is not representative of all SNAP recipients nationally or across SC. For example, in February 2026, SC SNAP recipients were 60% female, 39% White, 57.6% Black, and 94.1% non-Hispanic (16). Our sample was 100% female, 10.9% White, 80.0% Black, and 92.7% non-Hispanic. Our sampling approach allowed for rapid data collection within the current stage of the larger clinical trial; yet, it limits the generalizability of these findings and may bias results. While respondents were part of a larger clinical trial, it is unlikely this aspect biased the results, as participation in the parent trial did not alter benefit receipt or timing. Further, the survey items were developed by the research team and not previously validated or pilot tested. No established validated instruments were appropriate for the context of this event at the time of data collection, and the survey had to be developed and deployed quickly to capture information during a narrow and evolving window of relevance. The strengths of this study include the collection of important and timely data to document a first of its kind event.

Future research should explore the use of administrative or secondary data sources to further examine the impacts of the government shutdown in larger more representative sample of SNAP recipients. In conclusion, this study demonstrates the urgent need for contingency plans that ensure that continuity of SNAP benefits during federal disruptions to safeguard our nation’s most impactful safety net program and the families affected.

## Data Availability

The raw data supporting the conclusions of this article will be made available by the authors, without undue reservation.
